# Bio-oriented synthesis of new sulphadiazine derivatives for urease inhibition and their pharmacokinetic analysis

**DOI:** 10.1038/s41598-021-98413-x

**Published:** 2021-09-23

**Authors:** Asad Hamad, Mohsin Abbas Khan, Irshad Ahmad, Ruqaiya Khalil, Muhammad Khalid, Urva Abbas, Rahat Azhar, Jalal Uddin, Gaber El-Saber Batiha, Ajmal Khan, Zahid Shafiq, Ahmed Al-Harrasi

**Affiliations:** 1 Department of Pharmaceutical Chemistry, Faculty of Pharmacy, The Islamia University of Bahawalpur, Bahawalpur, 63100 Pakistan; 2 Dr. Panjwani Center for Molecular Medicine and Drug Research, International Center for Chemical and Biological Sciences, University of Karachi, Karachi, 75270 Pakistan; 3 Department of Chemistry, Khwaja Fareed University of Engineering and Information Technology, Rahim Yar Khan, 64200 Pakistan; 4 Institute of Chemical Sciences, Bahauddin Zakariya University, Multan, 60800 Pakistan; 5 Islam College of Pharmacy, Sialkot, Pakistan; 6 Department of Pharmaceutical Chemistry, College of Pharmacy, King Khalid University, Abha, 62529 Kingdom of Saudi Arabia; 7 Department of Pharmacology and Therapeutics, Faculty of Veterinary Medicine, Damanhour University, Damanhour, 22511 AlBeheira Egypt; 8 Natural and Medical Sciences Research Center, University of Nizwa, Birkat Al Mauz, P.O Box 33, 616 Nizwa, Oman

**Keywords:** Biochemistry, Chemical biology, Drug discovery

## Abstract

Current research is based on biology-oriented synthesis of sulphadiazine derivatives and determination of their urease inhibitory activity. In this regard, a series of (*E*)-4-(benzylideneamino)-*N*-(pyrimidin-2-yl)benzenesulfonamide was synthesized from sulphadiazine and substituted aromatic aldehydes. The structures of synthesized compounds were ascertained by spectroscopic techniques, such as, FTIR, NMR and HRMS analysis, and in-vitro and in-silico investigation were carried out for the inhibition of urease. Ureases are harmful for humans by producing by-products of urea (ammonia and carbon dioxide). The most active compound (**3l**) against urease exhibited IC_50_ value of 2.21 ± 0.45 µM which is 10 times more potent than the standard thiourea (20.03 ± 2.06 µM). It is noteworthy that most of our synthesized compounds showed significant to excellent activities against urease enzyme and most of them substituted by halogen or hydroxy groups at *ortho* and *para* positions in their structures. Inhibition of enzyme by the synthesized analogues was in descending order as 3l > 3a > 3b > 3q > 3e > 3o > 3s > 3t > 3g > 3k > 3r > 3f > 3m > 3p > 3n > 3j > 3i > 3h. Moreover, molecular docking studies were performed to rationalize the binding interactions of the synthesized motifs with the active pocket of the urease enzyme. The synthesized sulphadiazine derivatives (**3a–u**) were found to be non-toxic, and presented passive gastrointestinal absorption.

## Introduction

Ureases (EC 3.5.1.5) are similar to amidohydrolases and common in nature, being present in plants, microbes and animals^1^. In response to an external stimulus some organisms showed the gene for coding of urease similar to that of *Klebsiella* species, and some showed constitutively, for example *Bacillus pasteuri*^2^. All ureases have a common ancestral gene, as can be derived from the available data^3^. Since there is no difference in the sequence for plants and bacteria from the available data and they both have the same active sites, Jack bean urease was marked as a prototype for characterization and studying the urease enzymes^4^. Urease from Jack bean has a trimer—two structural subunits and one catalytic subunit. About 840 amino acids are present in active site-containing subunit and the catalytic mechanism requires Ni^2+^ in the active site^5^. Ureases also have great economic and medical importance. Many pathologic situations are endorsed by the ureases like peptic ulcer^6^, liver come, renal pathologies and urinary tract infections, encephalopathy of liver and pyelonephritis^7^. The action of urease on urea fertilizers increases ammonia production^8^ as well as pH of soil enhanced that causes crop damage. Therefore, adding urease inhibitors to urea fertilizers has remarkable effects on the removal of harmful effects of urea fertilizers^9^.

This synthesis of urease inhibitors is owing to their effects on pathology. There are four categories of urease inhibitors—thiolate compounds and hydroxamic acid, phosphoramidates and chelators of nickel atom at the active site. The fluoride ion is present in some types^10^, as well as hydroxamic acid that causes non-competitive inhibition of this enzyme. Thiols and phosphoramidates^11, 12^ are also said to be competitive inhibitors of this aforementioned enzyme^13^. Recently, the Schiff’s base derivatives^14^ are found to be the potent inhibitors of the urease from Jack bean. In this project, we determined inhibition of Jack bean^’^s urease by various (*E*)-4-(benzylideneamino)-*N*-(pyrimidin-2-yl)benzenesulfonamide (Schiff’s base derivatives of sulphadiazine). These moieties have a relation with a large group of sulfonamide derivatives and formed by the condensation of sulphadiazine with substituted aromatic aldehdyes. There are many biological effects shown by Schiff’s bases of sulphonamides attributed to the attached aldehydes^15^. Many chemotherapeutic properties given by the Schiff’s bases such as anti-malarial^16^, antimicrobial^17^, antifungal^18^, antiviral ^19^, anticonvulsant^20^ and antiplasmodial^21^, antioxidant^22^, enzymatic inhibition^23^ and catalytic activities^24^. By keeping in mind the above mentioned properties of Schiff’s bases, the biology-oriented drug synthesis was done via one step modification of sulphadiazine (**1**) and further screened for their diversified biological activities.

**Figure 1:**
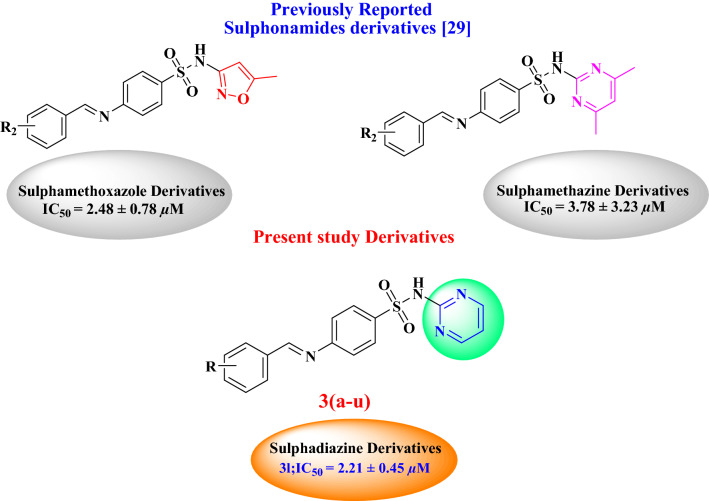
Rationale of present study with reported studies.

Previously we have reported several novel molecules with potent urease inhibition activity which belong to different functionalities^25–28^. Recently, we had incorporated Schiff’s base moiety with drugs of sulphonamide class and reported for urease inhibition potential^29^. Therefore, in search of more potent urease inhibitors, we have designed and synthesized Schiff’s base derivatives of marketed drug sulphadiazine (**1**) by using wide range of aromatic aldehydes (**2a–u**) and subjected them to urease inhibition followed by molecular docking, ADME analysis and structure activity relationship (SAR) studies to discuss the varying effects of different incorporated functional groups. The study concluded with potent urease inhibitors and an understanding of the structure activity relationship which would help to synthesize more potent scaffolds that may serve as potential drug candidates and can serve as active pharmaceutical ingredients. To the best of our knowledge, except compounds **3a, 3b, 3c, 3e, 3f, 3g, 3h, 3q** and **3t** all synthesized compounds are new (Fig. 1).

## Results and discussion

### Chemistry 

**Scheme 1:**
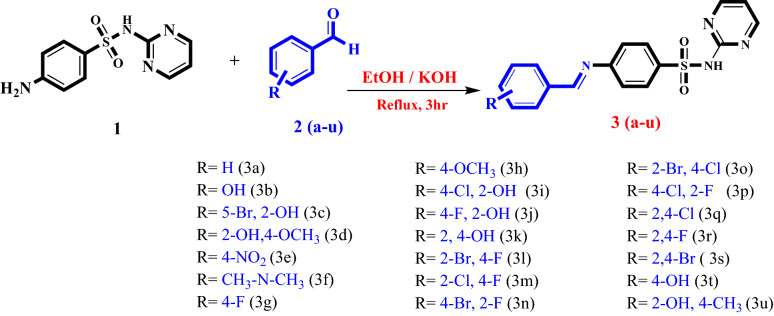
Synthesis of (*E*)-4-(benzylideneamino)-*N*-(pyrimidin-2-yl)benzenesulfonamide derivatives.

To explore the anti-urease potential of (*E*)-4-(benzylideneamino)-*N*-(pyrimidin-2-yl)benzenesulfonamide, Schiff’s base derivatives (**3a–u**) of sulphadiazine were synthesized through condensation of the sulphadiazine (**1**) with substituted aromatic aldehydes (**2a–u**). The reaction was performed by using KOH as basic catalyst. The most favorable conditions were attained by refluxing the reaction mixture in the presence of ethanol as solvent in the presence of potassium hydroxide (KOH) illustrated in Scheme 1. The scope of reaction was extended for synthesizing variety of substituted derivatives of sulphadiazine by using substituted (2 and 4 position) aryl aldehydes (**2a–u**).

The structures of the compounds were ascertained using different spectroscopic techniques. In ^1^H NMR of all compounds, proton of imine was in the range of δ 8.56 ppm to δ 8.96 ppm as a singlet. The spectral data of other aromatic protons are accordance with the structures of anticipated compounds and in ^13^C NMR of all compounds remarkable iminic carbon (C=N) peaks was in the range of δ 157.82–δ 162.11 ppm. In FTIR all the compounds showed C=N peak between 1643 cm^−1^ and 1697 cm^−1^ and no peak was observed for primary amine which was in complete accordance with the synthesized compounds. In HRMS all the compounds showed there [M+H]^+^ value which were in total agreement with the molecular weight of the synthesized compounds.

### Biology

All the synthesized derivatives were subjected to anti-urease activity. In preliminary screening, all compounds were evaluated at 1 mM concentrations, and percentage inhibition was used as an indicator of activity. IC_50_ values were calculated for the scaffolds having percentage inhibition higher than 50%. There was varying urease inhibition activity shown by the synthesized molecules and a range of compounds had better activity than thiourea. As it is evident from synthesized scaffolds, ranging from more active **3l** to having less activity **3h** relying on the functionalities attached with aromatic ring of aldehydes so bromine with fluorine showed the remarkable results. The IC_50_ values were in the order of **3l** (2.21 ± 0.45 µM) > **3a** (2.32 ± 0.54 µM) > **3b** (3.29 ± 2.00 µM) > **3q** (4.05 ± 1.10 µM) > **3e** (4.15 ± 0.77 µM) > **3o** (5.04 ± 0.4 µM) > **3s** (6.09 ± 0.45 µM) > **3t** (8.90 ± 2.45 µM) > **3g** (10.15 ± 0.45 µM) > **3k** (12.10 ± 2.43 µM) > **3r** (13.10 ± 2.55 µM) > **3f** (13.14 ± 0.02 µM) > **3m** (15.66 ± 2.45 µM) > **3p** (17.07 ± 0.02 µM) > **3n** (18.20 ± 1.78 µM) > **3j** (18.96 ± 1.23 µM) > **3i** (25.56 ± 0.22 µM) > **3h** (45.56 ± 0.56 µM) and rest of the compounds were not active. Structure–activity-relationship (SAR) studies of the synthesized compounds predicted that, in some cases, the nature, the position of varying groups attached to the benzene of aldehyde showed an effective role in the increase and decrease of urease inhibition^30^.

### Structure activity relationship studies

**Figure 2:**
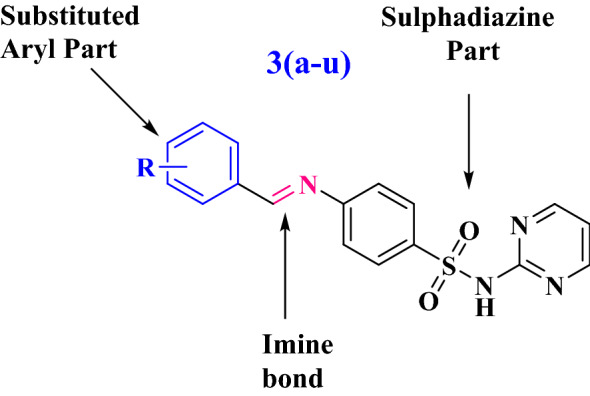
(*E*)-4-(benzylideneamino)-*N*-(pyrimidin-2-yl)benzenesulfonamide.

The synthesized derivatives of (*E*)-4-(benzylideneamino)-*N*-(pyrimidin-2-yl)benzenesulfonamide indicated that the substituted aldehydes used for Schiff’s base formation of drug have very diverse effect on the pocket of enzyme. To examine effects of electronic effects of substituents on activity, detailed study of SAR is mandatory. In order to rationalize our findings, the target molecules were divided into two hypothetical groups, one is sulphadiazine and other is aryl group with substituents of different electronic and steric effects at variable positions (Fig. 2).

**Figure 3:**
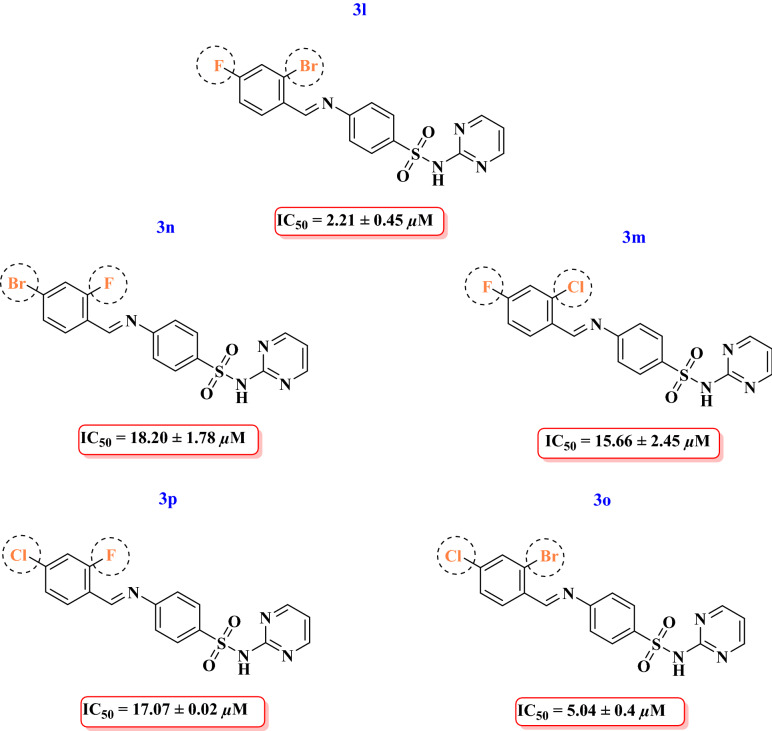
Structure–activity relationship of compounds **3o, 3p, 3l, 3m, 3n.**

Among all the multi halogenated Schiff’s base derivatives of sulphadiazine, compound **3l** having bromine at *ortho* and fluorine at *para* position of the attached aldehyde showed highest anti-urease activity (IC_50_ = 2.21 ± 0.45 µM) which is ten times more potent than the standard thiourea and nine time more potent than the compound **3n** having fluorine at *ortho* and bromine at *para* position. Moreover, compound **3m** having chlorine at *ortho* and flourine at *para* showed good potential (IC_50_ = 15.66 ± 2.45 µM) against urease might be due to high electron withdrawing capacity of the attached halogens whereas compound **3p** having fluorine at *ortho* and chlorine at *para* position showed decreased activity (IC_50_ = 17.07 ± 0.02 µM) as compared to 3m and good inhibitory activity as compared to standard thiourea. Replacement of fluorine with bromine in compound **3o** enhanced the inhibitory activity (IC_50_ = 5.04 ± 0.4 µM) as compared to 3p (Fig. 3).

**Figure 4:**
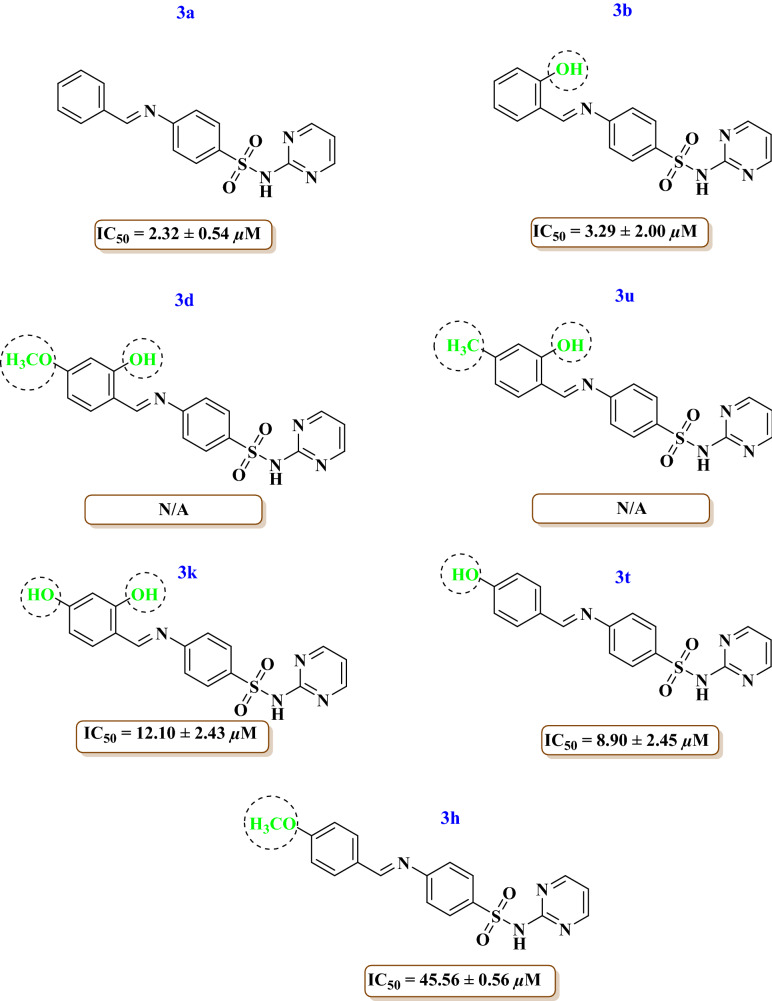
Structure–activity relationship of compounds **3a, 3b, 3d, 3h, 3k, 3t, 3u.**

Among the hydroxy, methyl and methoxy substituted derivatives, compound **3b** having hydroxy at *ortho* position showed significant activity (IC_50_ = 3.29 ± 2.00 µM) comparable with the compound **3a** (IC_50_ = 2.32 ± 0.54 µM) having unsubstituted aryl ring. Addition of methoxy at *para* with hydroxy at *ortho* in compound **3d** decreased the activity and replacement of methoxy with methyl in compound **3u** also decreased the activity as compared to 3b. Furthermore, addition of another hydroxy at para position in compound **3k** showed significant activity (IC_50_ = 12.10 ± 2.43 µM) higher than thiourea and less potent than 3b. Moreover, change of position of hydroxy from *ortho* to *para* in compound **3t** exhibited a good activity (IC_50_ = 8.90 ± 2.45 µM). Compound **3h** having methoxy at para position showed weak activity (IC_50_ = 45.56 ± 0.56 µM) (Fig. 4).

**Figure 5:**
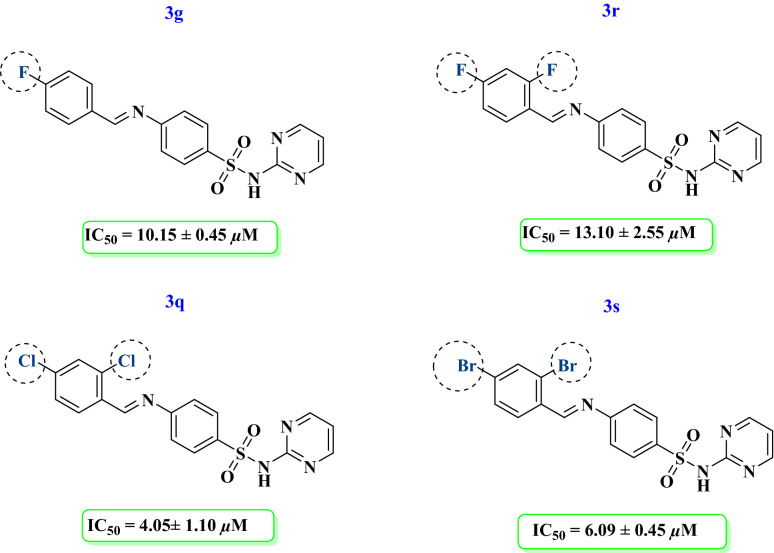
Structure–activity relationship of compounds **3g, 3q, 3r, 3s.**

Compound **3g** having fluorine at *para* position displayed significant activity (IC_50_ = 10.15 ± 0.45 µM) more potent than standard thiourea. Addition of another fluorine at *ortho* position in compound **3r** showed good activity (IC_50_ = 13.10 ± 2.55 µM). Compound **3s** having bromine at *ortho* and *para* position showed potent activity (IC_50_ = 6.09 ± 0.45 µM) against urease. Moreover, compound **3q** having chlorine at *ortho* and *para* position exhibited good activity (Fig. 5).

**Figure 6:**
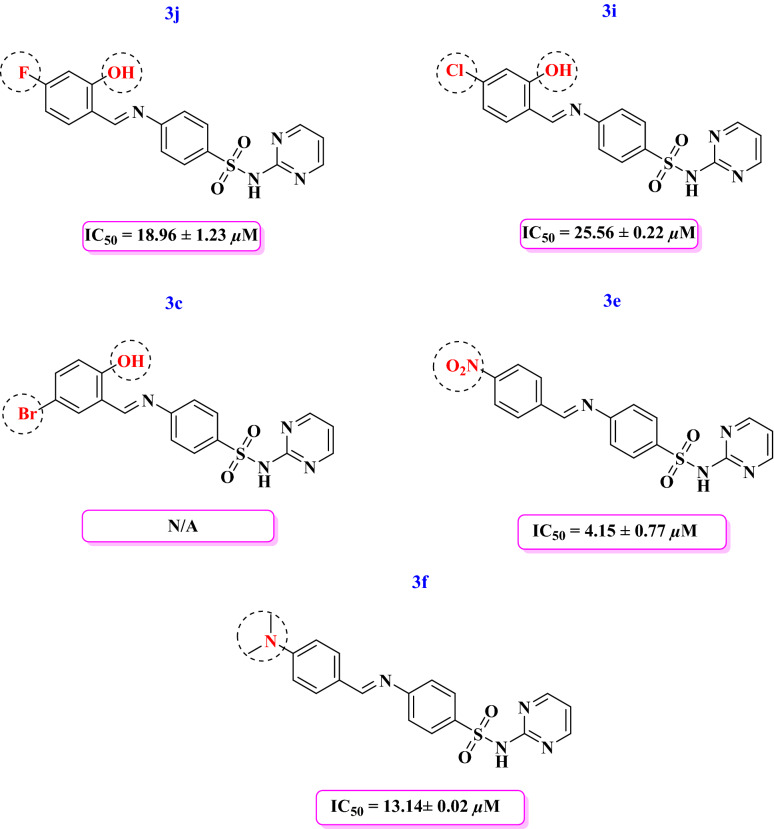
Structure–activity relationship of compounds **3c, 3e, 3f, 3i, 3j.**

Among the compounds containing hydroxy and halogen, compound **3j** having hydroxy at *ortho* and fluorine at *para* position exhibited good enzyme inhibitory activity (IC_50_ = 18.96 ± 1.23 µM). Replacement of fluorine with chlorine in compound **3i** decreased the activity (IC_50_ = 25.56 ± 0.22 µM) as compared to 3j. Further replacement of chlorine with bromine in compound **3c** caused the inactivation of the compound. Interestingly compound **3e** having nitro group at *para* position of the attached aldehyde showed a good activity (IC_50_ = 4.15 ± 0.77 µM). Another compound **3f** having dimethylamine at para position of the attached aryl ring showed a significant activity (IC_50_ = 13.14 ± 0.02 µM) (Fig. 6).

The above inhibitory activity evaluation demonstrated that the halogens have a good impact on the activity of the enzyme and this impact can alter as the position of the attached halogens changed on the phenyl ring of the synthesized derivatives. Moreover, the inhibitory activity assessment also exhibited that the nature and position of the other groups like hydroxy, methoxy, nitro and amino attached on the phenyl ring have varying effects on the activity of the enzyme. To further validate the effects of substituents on the activity of the enzyme, in silico analysis was performed on the synthesized derivatives.

### Molecular docking

**Figure 7:**
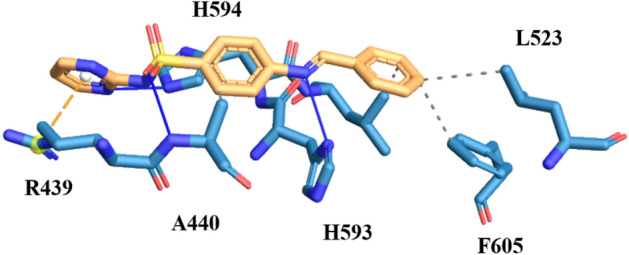
The simulated binding mode of compound **3a** in the binding pocket of Jack Bean Urease.

Structure activity relationship studies of sulphadiazine derivatives directed towards the *in-silico* screening of these derivatives, to check the binding affinity and activity of these synthesized compound at the active site of the urease enzyme. Amongst the most active derivatives of sulphadiazine, compound **3a**, having unsubstituted aryl ring came up as potential inhibitor under in-vitro and in-silico analysis by exhibiting IC_50_ value of 2.32 ± 0.54 µM and strong hydrogen bonding, hydrophobic and π-cation interactions, respectively. The oxygen of the sulphonamide moiety formed hydrogen boding with the nitrogen of the A440 (distance 3.55 Å). Moreover, the imidazole ring of H593 interacting through the hydrogen bonding with the nitrogen of the Schiff base at the distance of 2.38 Å and nitrogen of pyridazine interacting with the imidazole ring of H594 (distance 2.58 Å) through hydrogen bonding. Apart from these interactions, compound 61 bind with the enzyme through hydrophobic contacts with A440, L523, L525 and F605. Further insights revealed that the compound also exhibiting π-cation interaction with amino acid R439 (Fig. 7).

**Figure 8:**
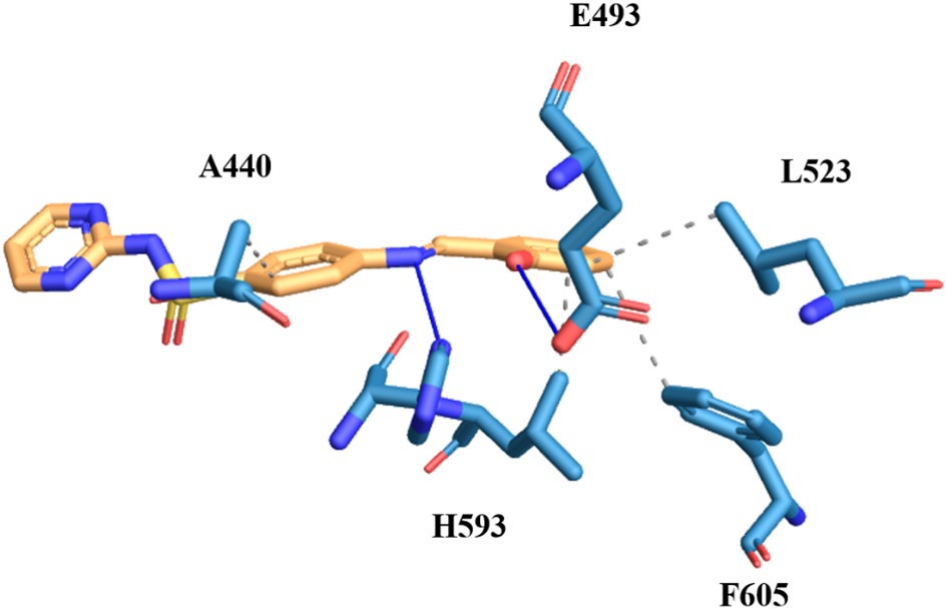
The simulated binding mode of compound **3b** in the binding pocket of Jack Bean Urease.

Compound **3b**, which has hydroxyl at *ortho* position of the aryl ring, displayed strong hydrogen bonding between oxygen of the amino acid E493 and hydroxyl group of the attached aryl ring at the distance of 3.33 Å. Compound also showed interaction between side chain of the imidazole ring of H593 and nitrogen of the imine through hydrogen bonding (distance 2.36 Å). Further insights exhibited that the compound also bind with urease amino acids through hydrophobic interaction among A440, L523, L595 and F605 at the distances of 3.41 Å, 3.87 Å, 3.61 Å and 3.95 Å, respectively. The binding modes of this compound are showed in Fig. 8.

**Figure 9:**
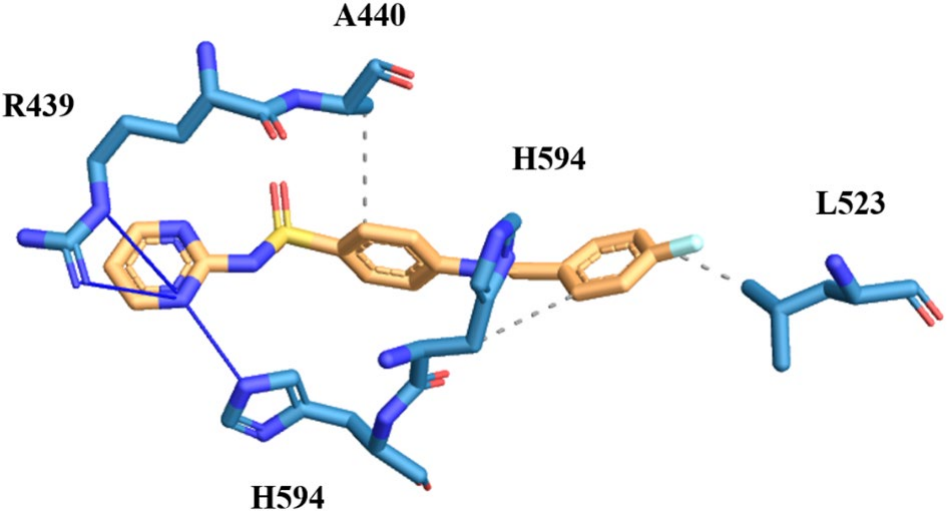
The simulated binding mode of compound **3g** in the binding pocket of Jack Bean Urease.

Compound **3g**, having fluorine at *para* position of the aryl ring exhibited hydrogen bonding and apolar interactions. In this compound the nitrogen of the pyridazine ring formed hydrogen bonding with the R439 at the distances 3.70 Å and 3.71 Å and at the same time nitrogen of pyridazine ring also formed hydrogen bond with the imidazole ring of the H94. The nitrogen of the imine bond also interacting with imidazole ring of the amino acid H593 through hydrogen bonding. Apart from such contact, compound 3g also interacting with amino acids A440, L523 and H593 through hydrophobic interaction. From the aforementioned interactions, it can be concluded that this compound is potentially active against urease enzyme by exhibiting good *in-vitro* and in-silico activities (Fig. 9).

**Figure 10:**
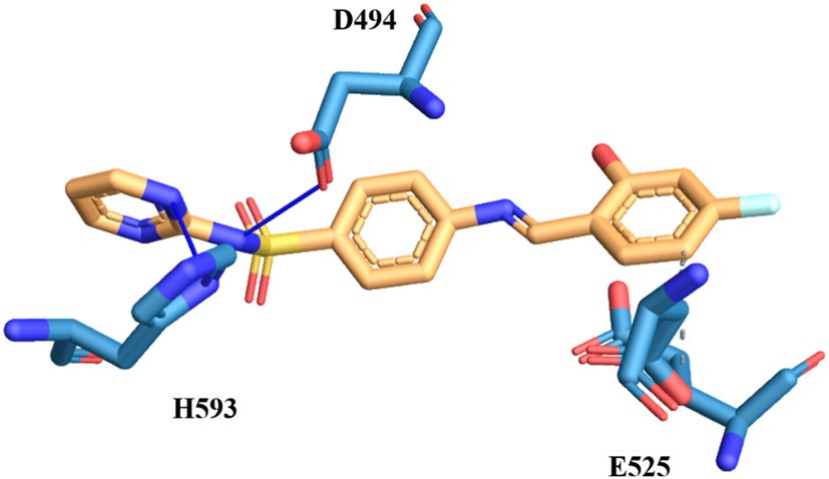
The simulated binding mode of compound **3j** in the binding pocket of Jack Bean Urease.

Compound **3j**, having hydroxy at *ortho* and fluorine at *para* position of the aromatic ring exhibited apolar and hydrogen bond interactions. The nitrogen of the sulfonamide moiety interacting with the amino acid D494 and nitrogen of the pyridazine ring interacting with the side chain of H593. Besides these interactions, compound 3j also interacting through hydrophobic interactions with amino acids D521 and E525. Thus from molecular docking studies of this compound, it can be concluded that it can serve as potent urease inhibitor Fig. 10.

By analyzing the docking data it can be deduced that the experimental and docking results have a coherence among each other. Moreover, molecular docking results guided us to the way that the nature, position and quantity of the attached substituted groups on the aryl ring can affect the activity of the enzyme. Furthermore, it can be seen from the interactions in in-silico analyses that the activity of the enzyme is inhibited by different binding poses of all the potent compounds.

### Pharmacokinetic studies

**Figure 11:**
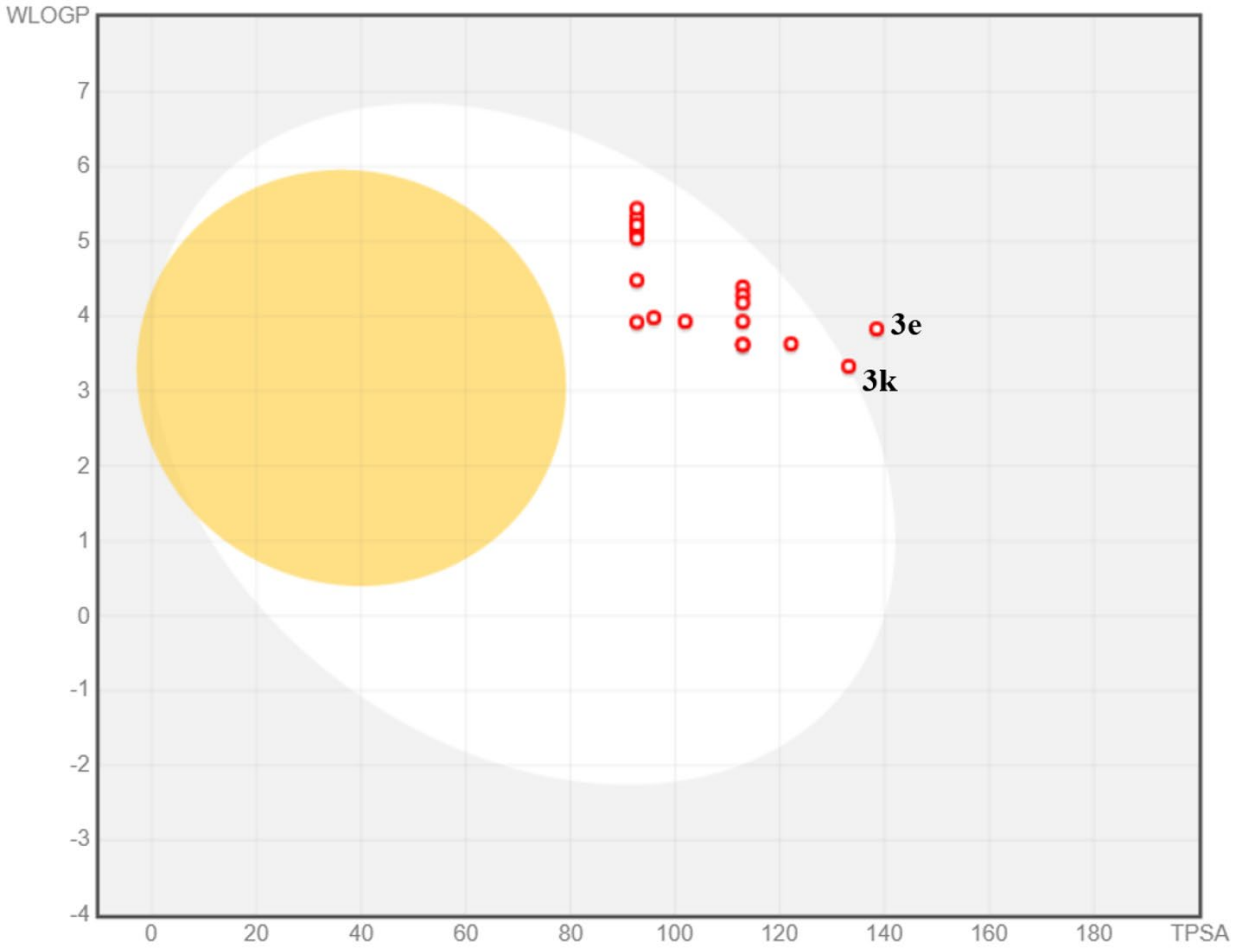
The boiled-egg plot of the synthesized derivatives **3(a–u)**.

In-silico analysis is another way for the estimation of drug likeness, physicochemical and pharmaceutical properties of the synthesized derivatives. The computer guided study of the pharmacokinetic parameters provides the rational way to evaluate the molecules by decreasing the associated cost and labor in in-vitro experimentation. So for this purpose the ADME parameters were analyzed for the synthesized molecules **3(a–u)** using a web based SwissADME. For assessing the passive gastrointestinal absorption of the synthesized compounds, the boiled-egg plot produced in SwissADME is a fast and a good methodology^31, 32^. The white part of the egg representing the gastrointestinal absorption of compounds and the yellow inner yolk of the egg representing the compounds which can cross the blood brain barrier and the produced data is summarized in Table 1. From the graph it can be seen that the all the molecules exhibited high GI absorption while compound **3k** and **3e** showed low GI absorption furthermore none of the moieties displayed brain permeation (Fig. 11).

**Table 1:** In-silico ADME evaluation of synthesized compounds (3a-u).

Compound	TPSA^a^	LV^b^	PAINS^c^	WLOGP^d^	AHA^e^	BBB^f^	GIA^g^	LL^h^	BA^i^
**3a**	92.69	0	0	3.92	18	No	High	0	0.55
**3b**	112.92	0	0	3.62	18	No	High	1	0.55
**3c**	112.92	0	0	4.39	18	No	High	1	0.55
**3d**	122.15	0	0	3.63	18	No	High	1	0.55
**3e**	138.51	0	0	3.83	18	No	Low	1	0.55
**3f**	95.93	0	0	3.98	18	No	High	1	0.55
**3g**	92.69	0	0	4.48	18	No	High	1	0.55
**3h**	101.92	0	0	3.93	18	No	High	1	0.55
**3i**	112.92	0	0	4.28	18	No	High	1	0.55
**3j**	112.92	0	0	4.18	18	No	High	1	0.55
**3k**	133.15	0	0	3.33	18	No	Low	1	0.55
**3l**	92.69	0	0	5.24	18	No	High	1	0.55
**3m**	92.69	0	0	5.13	18	No	High	1	0.55
**3n**	92.69	0	0	5.24	18	No	High	1	0.55
**3o**	92.69	0	0	5.33	18	No	High	2	0.55
**3p**	92.69	0	0	5.13	18	No	High	1	0.55
**3q**	92.69	0	0	5.22	18	No	High	2	0.55
**3r**	92.69	0	0	5.04	18	No	High	1	0.55
**3s**	92.69	0	0	5.44	18	No	High	2	0.55
**3t**	112.92	0	0	3.62	18	No	High	1	0.55
**3u**	112.92	0	0	3.93	18	No	High	1	0.55

^a^Topological polar surface area, ^b^Lipinski violations, ^c^pan-assay interference, ^d^logarithm of partition coefficient between n-octanol and water, ^e^aromatic heavy atom, ^f^blood brain barrier permeation, ^g^gastrointestinal absorption, ^h^lead likeness, ^i^bioavailability.

All the molecules displayed high gastrointestinal absorption except two molecules and all the moieties showed good results for TPSA (topological surface area) in the range of 92.69–138.51 Å. Moreover, all the scaffolds are complying with the Lipinski’s rule of five^33^. Furthermore, PAINS filter was applied over the synthesized molecules^34^ and the obtained data reflected that the synthesized compounds are novel and they do not show any similarity with the PAINS. Thus from the obtained data it can be suggested that these synthesized moieties can serve as a novel compounds for the anti-urease drug design.

## Conclusions

In the current study, we synthesized and screened a series of (*E*)-4-(benzylideneamino)-*N*-(pyrimidin-2-yl)benzenesulfonamide for their anti-urease activity. Derivative **3l** having IC_50_ = 2.22 ± 0.45 µM was among the most active inhibitors of Jack bean enzyme and more active than the standard inhibitor. As these compounds have structural similarity with basic skeleton of urease substrate, they showed good activity. Therefore, in future drug development, these moieties could be used as template for designing more biologically active derivatives through modifications or derivatization.

## Experimental section

### Chemistry

#### General procedure for the synthesis of (*E*)-4-(benzylideneamino)-*N*-(pyrimidin-2-yl)benzenesulfonamides 3(a–u)

To a stirred solution of sulphadiazine (**1,** 0.1 mmol) in absolute ethanol (10 ml) was added appropriate aldehyde (**2a–u,** 0.1 mmol). Methanolic KOH (0.01 mmol) was added in catalytic amount and the resulted mixture was heated under reflux for 3h (Scheme 1). The course of the reaction was monitored by TLC (EtOAc: Pet. ether 1:1). After the completion of the reaction, the crystalline solid formed was filtered and washed 3 times with hot ethanol and dried under vacuum to give the sulfonamides (**3a–u**) in good to excellent yields (76–86%). The products obtained were recrystallized in methanol.

### Materials and methods

All the solvents and reagents are of high purity and have been purchased commercially from Sigma-Aldrich, Fluorochem, Alfa Aesar and Fisher Scientific. Melting points were recorded using a Gallen kamp melting point apparatus and are uncorrected. ^1^H and ^13^C NMR analyses were performed on a 400 MHz spectrometer (Bruker) equipped with a SampleXpress (from Bruker) auto sampler system, using deuterated solvents for the preparation of the samples. The obtained spectra were analyzed using Topspin 7.1 software (Bruker). The chemical shifts were reported in ppm values relative to tetramethylsilane (TMS), used as internal standard. Signals were identified and described as singlet (s), doublet (d), triplet (t) and multiplet (m). Coupling constants were reported in Hertz (Hz). Mass Spectra (ESI), HRMS Agilent technologies 6890N and an inert mass selective detector 5973 mass spectrometer Technologies. Glassware used was dried in a UN55 oven (Memmert) at 200 °C^35^. Fourier transform Infrared (FTIR) spectrum of the synthesized compounds was taken using KBr pellet press method by Bruker TENSOR 27 FTIR spectrophotometer and the (%) yields are calculated on the basis of 1.0 mM of each reactant used. The synthesized motifs **3(a–u)** were characterized as given below:

#### 4-(Benzylideneamino)-*N*-(pyrimidin-2-yl)benzenesulfonamide (3a)^36^

Physical appearance: orange red crystals; yield (76%); m. p. 269–271 °C.

#### 4-(2-Hydroxybenzylideneamino)-*N*-(pyrimidin-2-yl)benzenesulfonamide (3b)^37^

Physical appearance: yellow crystals; yield (78%), m. p. 277–279 °C.

#### 4-(5-Bromo-2-hydroxybenzylideneamino)-*N*-(pyrimidin-2-yl)benzenesulfonamide (3c)^37^

Physical appearance: orange red crystals; yield (81%); m. p. 282–284 °C.

#### 4-(2-Hydroxy-4-methoxybenzylideneamino)-*N*-(pyrimidin-2-yl)benzenesulfonamide (3d)

Physical appearance: yellow crystals; yield (82%); m. p. 266–267 °C; FTIR (KBr, cm^−1^), 3326 (NH), 2977, 2875 (CH), 1696 (C=N), 1568 (CH=CH), 1463 (C−N), 1397 (C−O), 1192 (SO_2_); ^1^H NMR (400 MHz, DMSO-*d*_*6*_), δ ppm; 3.38 (s, 1H, NH), 3.85 (s, 3H, Ar–OCH_3_), 6.62 (s, 1H, Ar–H), 6.67 (d, 1H, *J* = 7.08 Hz, Ar–H), 7.03 (t, 1H, *J* = 7.32 Hz, Pym–H), 7.39 (d, 2H, *J* = 8.67 Hz, Ar–H), 7.71 (d, 1H, *J* = 7.33 Hz, Ar–H), 7.79 (d, 2H, *J* = 8.19 Hz, Ar–H), 8.48 (d, 2H, *J* = 7.32 Hz, Pym–H), 8.56 (s, 1H, HC=N), 9.78(s,1H, Ar–OH); ^13^C NMR (400 MHz, DMSO-*d*_*6*_), δ ppm; 55.97, 103.43, 107.18, 113.14, 115.86, 124.78, 129.56, 133.80, 138.21, 157.67, 158.72, 160.21, 162.36, 166.34, 168.78; HRMS (ESI, *m/z*) (%): calculated C_18_H_16_ N_4_O_4_S: 384.09 found [M+H]^+^ 385.09 (100).

#### 4-(4-Nitrobenzylideneamino)-*N*-(pyrimidin-2-yl)benzenesulfonamide (3e)^38^

Physical appearance: orange red crystals; yield (83%); m. p. 274–276 °C.

#### 4-(4-(Dimethylamino)benzylideneamino)-*N*-(pyrimidin-2-yl)benzenesulfonamide (3f)

Physical appearance: orange crystals; yield (84%), m. p. 275–277 °C.

#### 4-(4-Fluorobenzylideneamino)-*N*-(pyrimidin-2-yl)benzenesulfonamide (3g)^39^

Physical appearance: light red crystals; yield (77%); m. p. 283–285 °C.

#### 4-(4-Methoxybenzylideneamino)-*N*-(pyrimidin-2-yl)benzenesulfonamide (3h)

Physical appearance: light yellow crystals; yield (86%); m. p. 287–289 °C.

#### 4-(4-Chloro-2-hydroxybenzylideneamino)-*N*-(pyrimidin-2-yl)benzenesulfonamide (3i)

Physical appearance: orange red crystals; yield (80%), m. p. 285–287 °C; FTIR (KBr, cm^−1^), 3383 (NH), 2881, 2983 (CH), 1691 (C=N), 1587 (CH=CH), 1475 (C−N), 1329 (C−O), 1146 (SO_2_); ^1^H NMR (400 MHz, DMSO-*d*_*6*_), δ ppm; 6.01 (s, 1H, Ar–H), 6.55 (d, 1H, *J* = 7.91 Hz, Ar–H), 7.02 (t, 1H, *J* = 7.02 Hz, Pym–H), 7.53 (d, 2H, *J* = 8.29 Hz, Ar–H), 7.61 (d, 1H, *J* = 7.15 Hz, Ar–H), 7.91 (d, 2H, *J* = 8.37 Hz, Ar–H), 8.47 (d, 2H, *J* = 7.32 Hz, Pym–H), 8.96 (s, 1H, HC=N), 11.86 (s,1H, Ar–OH), 12.84 (s, 1H, NH); ^13^C NMR (400 MHz, DMSO-*d*_*6*_), δ ppm; 115.98, 116.31, 118.97, 120.16, 122.21, 129.55, 134.09, 138.61, 152.47, 157.32, 157.69, 161.46, 164.53, 168.79; HRMS (ESI, *m/z*) (%): calculated C_17_H_13_ ClN_4_O_3_S: 388.04 found [M+H]^+^ 389.08 (100).

#### 4-(4-Fluoro-2-hydroxybenzylideneamino)-*N*-(pyrimidin-2-yl)benzenesulfonamide (3j)

Physical appearance: orange red crystals; yield (79%); m. p. 279–281 °C; FTIR (KBr, cm^−1^), 3357 (NH), 2805, 2880, 2980 (CH), 1697 (C=N), 1580 (CH=CH), 1477 (C−N), 1336 (C−O), 1138 (SO_2_); ^1^H NMR (400 MHz, DMSO-*d*_*6*_), δ ppm; 6.01 (s, 1H, Ar–H), 6.56 (d, 1H, *J* = 7.01 Hz, Ar–H), 7.00 (t, 1H, *J* = 7.12 Hz, Pym–H), 7.53 (d, 2H, *J* = 8.69 Hz, Ar–H), 7.61 (d, 1H, *J* = 7.35 Hz, Ar–H), 8.04 (d, 2H, *J* = 8.31 Hz, Ar–H), 8.48 (d, 2H, *J* = 7.02 Hz, Pym–H), 8.97 (s, 1H, HC=N), 10.19 (s,1H, Ar–OH), 13.11(s, 1H, NH); ^13^C NMR (400 MHz, DMSO-*d*_*6*_), δ ppm; 104.01, 107.53, 115.99, 117.00, 122.18, 129.56, 135.29, 138.50, 157.33, 157.70, 162.96, 163.10, 164.90, 167.13; HRMS (ESI, *m/z*) (%): calculated C_17_H_13_ FN_4_O_3_S: 372.07 found [M+H]^+^ 373.07 (100).

#### 4-(2,4-Dihydroxybenzylideneamino)-*N*-(pyrimidin-2-yl)benzenesulfonamide (3k)

Physical appearance: orange red crystals; yield (81%); m. p. 280–282 °C; FTIR (KBr, cm^−1^), 3324 (NH), 2805, 2882 (CH), 1688 (C=N), 1587 (CH=CH), 1468 (C−N), 1337 (C−O), 1149 (SO_2_); ^1^H NMR (400 MHz, DMSO-*d*_*6*_), δ ppm; 6.41 (d, 1H, *J* = 7.51 Hz, Ar–H), 7.05 (t, 1H, *J* = 7.65 Hz, Pym–H), 7.49 (s, 1H, Ar–H), 7.52 (d, 2H, *J* = 8.09 Hz, Ar–H), 7.64 (d, 1H, *J* = 7.98 Hz, Ar–H), 8.01 (d, 2H, *J* = 8.71 Hz, Ar–H), 8.48 (d, 2H, *J* = 7.77 Hz, Pym–H), 8.83 (s, 1H, HC=N), 9.93(s,1H, Ar–OH), 10.64(s,1H, Ar–OH), 10.92(s, 1H, NH); ^13^C NMR (400 MHz, DMSO-*d*_*6*_), δ ppm; 102.84, 108.87, 112.63, 115.97, 121.89, 129.57, 133.24, 137.77, 157.33, 158.70, 160.71, 163.71, 164.84, 165.63; HRMS (ESI, *m/z*) (%): calculated C_17_H_14_N_4_O_4_S: 370.07 found [M+H]^+^ 371.07 (100).

### 4-(2-Bromo-4-fluorobenzylideneamino)-*N*-(pyrimidin-2-yl)benzenesulfonamide (3l)

Physical appearance: orange red crystals; yield (82%), m. p. 285–287 °C; FTIR (KBr, cm^−1^), 3342 (NH), 2975 (CH), 1653 (C=N), 1586 (CH=CH), 1424 (C−N), 1332 (C−O), 1143 (SO_2_); ^1^H NMR (400 MHz, DMSO-*d*_*6*_), δ ppm; 6.39 (t, 1H, *J* = 7.95 Hz, Pym–H), 7.22 (s, 1H, Ar–H), 7.45 (d, 1H, *J* = 8.34 Hz, Ar–H), 7.53 (d, 2H, *J* = 8.19 Hz, Ar–H), 7.75 (d, 1H, *J* = 7.87 Hz, Ar–H), 7.88 (d, 2H, *J* = 8.51 Hz, Ar–H), 8.21 (d, 2H, *J* = 7.17 Hz, Pym–H), 8.73 (s, 1H, HC=N), 10.19 (s, 1H, NH); ^13^C NMR (400 MHz, DMSO-*d*_*6*_), δ ppm; 102.70, 112.29, 116.21, 120.94, 126.34, 128.58, 131.23, 133.23, 138.47, 157.35, 157.56, 165.03, 166.04; HRMS (ESI, *m/z*) (%): calculated C_17_H_12_ BrFN_4_O_2_S: 433.98 found [M+H]^+^ 434.98 (100).

#### 4-(2-Chloro-4-fluorobenzylideneamino)-*N*-(pyrimidin-2-yl)benzenesulfonamide (3m)

Physical appearance: orange red crystals; yield (78%); m.p. 277–279 °C; FTIR (KBr, cm^−1^), 3355 (NH), 2806, 2879, 2977 (CH), 1643 (C=N), 1580 (CH=CH), 1496 (C−N), 1331 (C−O), 1134 (SO_2_); ^1^H NMR (400 MHz, DMSO-*d*_*6*_), δ ppm; 6.74 (s, 1H, Ar–H), 7.42 (t, 1H, *J* = 7.75 Hz, Pym–H), 7.64 (d, 1H, *J* = 8.04 Hz, Ar–H), 7.81 (d, 2H, *J* = 8.23 Hz, Ar–H), 7.85 (d, 1H, *J* = 7.07 Hz, Ar–H), 8.04 (d, 2H, *J* = 8.21 Hz, Ar–H), 8.77 (d, 2H, *J* = 7.66 Hz, Pym–H), 8.70 (s, 1H, HC=N), 10.10 (s, 1H, NH); ^13^C NMR (400 MHz, DMSO-*d*_*6*_), δ ppm; 112.58, 115.42, 117.95, 120.77, 128.77, 130.16, 136.68, 136.79, 144.14, 156.61, 157.82, 158.27, 165.44, 166.24; HRMS (ESI, *m/z*) (%): calculated C_17_H_12_ FClN_4_O_2_S: 390.04 found [M+H]^+^ 391.04 (100).

#### 4-(4-Bromo-2-fluorobenzylideneamino)-*N*-(pyrimidin-2-yl)benzenesulfonamide (3n)

Physical appearance: orange red crystals; yield (80%); m.p. 291–293 °C; FTIR (KBr, cm^−1^), 3345 (NH), 2806 (CH), 1654 (C=N), 1576 (CH=CH), 1410 (C−N), 1327 (C−O), 1135 (SO_2_); ^1^H NMR (400 MHz, DMSO-*d*_*6*_), δ ppm; 6.39 (t, 1H, *J* = 7.34 Hz, Pym–H), 6.42 (s, 1H, Ar–H), 7.25(d, 2H, *J* = 8.45 Hz, Ar–H), 7.56 (d, 1H, *J* = 8.64 Hz, Ar–H), 7.72 (d, 1H, *J* = 7.79 Hz, Ar–H), 7.84(d, 2H, *J* = 8.09 Hz, Ar–H), 8.12 (d, 2H, *J* = 7.63 Hz, Pym–H), 8.71 (s, 1H, HC=N), 10.17(s, 1H, NH); ^13^C NMR (400 MHz, DMSO-*d*_*6*_), δ ppm; 115.43, 120.00, 120.55, 123.25, 126.03, 128.51, 128.78, 133.32, 139.23, 153.54, 157.57, 160.92, 163.50, 164.96; HRMS (ESI, *m/z*) (%): calculated C_17_H_12_ BrFN_4_O_2_S: 433.98 found [M+H]^+^ 434.98 (100).

#### 4-(2-Bromo-4-chlorobenzylideneamino)-*N*-(pyrimidin-2-yl)benzenesulfonamide (3o)

Physical appearance: orange red crystals; yield (76%); m.p. 277–279 °C; FT-IR (cm^−1^), 3383 (NH), 2803, 2882, 2977 (CH), 1654 (C=N), 1589 (CH=CH), 1474 (C−N), 1335 (C−O), 1149 (SO_2_); ^1^H NMR (400 MHz, DMSO-*d*_*6*_), δ ppm; 6.39 (t, 1H, *J* = 7.09 Hz, Pym–H), 7.25(d, 2H, *J* = 8.69 Hz, Ar–H), 7.45 (d, 1H, *J* = 8.02 Hz, Ar–H), 7.67 (d, 1H, *J* = 7.28 Hz, Ar–H), 7.87 (s, 1H, Ar–H), 7.98(d, 2H, *J* = 8.13 Hz, Ar–H), 8.49 (d, 2H, *J* = 7.91 Hz, Pym–H), 8.73 (s, 1H, HC=N), 10.16(s, 1H, NH); ^13^C NMR (400 MHz, DMSO-*d*_*6*_), δ ppm; 112.29, 120.54, 126.28, 128.59, 128.68, 130.48, 133.02, 133.39, 137.43, 138.09, 151.64, 157.57, 158.52, 165.04; HRMS (ESI, *m/z*) (%): calculated C_17_H_12_ BrClN_4_O_2_S: 449.96 found [M+H]^+^ 450.96 (100).

#### 4-(4-Chloro-2-fluorobenzylideneamino)-*N*-(pyrimidin-2-yl)benzenesulfonamide (3p)

Physical appearance: orange red crystals; yield (82%); m. p. 275–277 °C; FTIR (KBr, cm^−1^), 3430 (NH), 2803, 2882, 2985 (CH), 1656 (C=N), 1579 (CH=CH), 1347 (C−O), 1474 (C−N), 1127 (SO_2_); ^1^H NMR (400 MHz, DMSO-*d*_*6*_), δ ppm; 6.41 (t, 1H, *J* = 7.09 Hz, Pym–H), 6.79 (s, 1H, Ar–H), 7.26(d, 1H, *J* = 8.34 Hz, Ar–H), 7.43 (d, 2H, *J* = 8.18 Hz, Ar–H), 7.83 (d, 1H, *J* = 7.01 Hz, Ar–H), 8.09(d, 2H, *J* = 8.67 Hz, Ar–H), 8.36 (d, 2H, *J* = 7.85 Hz, Pym–H), 8.73 (s, 1H, HC=N), 10.17(s, 1H, NH); ^13^C NMR (400 MHz, DMSO-*d*_*6*_), δ ppm; 117.23, 117.48, 120.61, 122.92, 125.90, 128.55, 129.77, 137.59, 137.70, 153.55, 157.59, 161.07, 163.62, 164.66; HRMS (ESI, *m/z*) (%): calculated C_17_H_12_ FClN_4_O_2_S: 390.04 found [M+H]^+^ 391.04 (100).

#### 4-(2,4-Dichlorobenzylideneamino)-*N*-(pyrimidin-2-yl)benzenesulfonamide (3q)^38^

Physical appearance: orange red crystals; yield (81%); m. p. 291–293 °C.

#### 4-(2,4-Difluorobenzylideneamino)-*N*-(pyrimidin-2-yl)benzenesulfonamide (3r)

Physical appearance: orange red crystals; yield (81%); m. p.279–281 °C; FTIR (KBr, cm^−1^), 3358 (NH), 2806, 2880, 2976 (CH), 1696 (C=N), 1589 (CH=CH), 1498 (C−N), 1415 (C−O), 1199 (SO_2_); ^1^H NMR (400 MHz, DMSO-*d*_*6*_), δ ppm; 6.63 (s, 1H, Ar–H), 6.97 (t, 1H, *J* = 7.04 Hz, Pym–H), 7.29 (d, 1H, *J* = 8.53 Hz, Ar–H), 7.60 (d, 2H, *J* = 8.44 Hz, Ar–H), 7.89 (d, 1H, *J* = 7.47 Hz, Ar–H), 8.24(d, 2H, *J* = 8.78 Hz, Ar–H), 8.46 (d, 2H, *J* = 7.29 Hz, Pym–H), 8.71 (s, 1H, HC=N), 10.17(s, 1H, NH); ^13^C NMR (400 MHz, DMSO-*d*_*6*_), δ ppm; 112.10, 112.58, 113.27, 115.67, 120.90, 128.83, 130.42, 143.03, 153.15, 157.98, 161.69, 161.82, 164.24, 164.36; HRMS (ESI, *m/z*) (%): calculated C_17_H_12_ F_2_N_4_O_2_S: 374.06 found [M+H]^+^ 375.06 (100).

#### 4-(2,4-Dibromobenzylideneamino)-*N*-(pyrimidin-2-yl)benzenesulfonamide (3s)

Physical appearance: orange red crystals; yield (77%); m. p. 273–275 °C; FTIR (KBr, cm^−1^), 3387 (NH), 2880, 2987 (CH), 1653 (C=N), 1574 (CH=CH), 1402 (C−N), 1329 (C−O), 1156 (SO_2_); ^1^H NMR (400 MHz, DMSO-*d*_*6*_), δ ppm; 7.01 (t, 1H, *J* = 7.43 Hz, Pym–H), 7.36 (d, 2H, *J* = 8.76 Hz, Ar–H), 7.61 (d, 1H, *J* = 8.19 Hz, Ar–H), 7.75 (d, 1H, *J* = 7.17 Hz, Ar–H), 7.97(d, 2H, *J* = 8.37 Hz, Ar–H), 8.12 (s, 1H, Ar–H), 8.47 (d, 2H, *J* = 7.96 Hz, Pym–H), 8.72 (s, 1H, HC=N), 10.17 (s, 1H, NH); ^13^C NMR (400 MHz, DMSO-*d*_*6*_), δ ppm; 115.92, 121.34, 126.57, 126.66, 129.25, 130.80, 132.15, 132.84, 135.78, 136.44, 153.47, 157.77, 160.36, 164.36; HRMS (ESI, *m/z*) (%): calculated C_17_H_12_ Br_2_N_4_O_2_S: 493.90 found [M+H]^+^ 494.90 (100).

#### 4-(4-Hydroxybenzylideneamino)-*N*-(pyrimidin-2-yl)benzenesulfonamide (3t)^36^

Physical appearance: light orange crystals; yield (74%); m. p. 271–273 °C.

#### 4-(2-Hydroxy-4-methylbenzylideneamino)-*N*-(pyrimidin-2-yl)benzenesulfonamide (3u)

Physical appearance: yellowish red crystals; yield (86%); m. p. 279–281 °C; FTIR (KBr, cm^−1^), 3345 (NH), 2877 (CH), 1649 (C=N), 1575 (CH=CH), 1487 (C−N), 1354 (C−O), 1130 (SO_2_); ^1^H NMR (400 MHz, DMSO-*d*_*6*_), δ ppm; 2.30 (s, 3H, Ar–CH_3_), 5.96 (s, 1H, NH), 6.78 (s, 1H, Ar–H), 6.82 (d, 1H, *J* = 8.35 Hz, Ar–H), 6.97(t, 1H, *J* = 7.18 Hz, Pym–H), 7.39 (d, 2H, *J* = 8.65 Hz, Ar–H), 7.60 (d, 1H, *J* = 8.06 Hz, Ar–H), 7.90 (d, 2H, *J* = 7.36 Hz, Ar–H), 8.45 (d, 2H, *J* = 7.19 Hz, Pym–H), 8.92 (s, 1H, HC=N), 10.17(s, 1H, Ar–OH); ^13^C NMR (400 MHz, DMSO-*d*_*6*_), δ ppm; 115.57, 117.43, 120.52, 121.08, 121.17, 128.87, 130.19, 144.82, 148.01, 153.30, 157.86, 160.87, 161.33, 164.45; HRMS (ESI, *m/z*) (%): calculated C_18_H_16_N_4_O_3_S: 368.09 found [M+H]^+^ 369.09 (100).

### Urease activity of synthesized moieties

Indophenol method was used to perform the urease inhibition^40^. 10 µL of enzyme, 40 µL buffer, 10 µL of assay moiety, 40-µL each of phenol-reagents, and alkali reagent were present in the assay mixture. Firstly, incubation of assay compounds and enzyme was done in assay buffer at 37 °C for 30 min. Then, alkaline reagents and 40 µL of phenol were mixed to quench the mixture. 96 well plates were used in enzyme assay and for reading of absorbance at 625 nm a microplate reader was helpful. The experiment was done in triplicate and PRISM 4.0 was used to get IC_50_, positive control was thiourea.

#### Molecular docking studies

Molecular docking studies were carried out to investigate the molecular basis of the resulted urease inhibition demonstrated by the newly synthesized benzenesulfonamide derivatives. In this connection, the crystal structure of the Jack Bean (*Canavalia ensiformis*) Urease under the accession code 4GY7 was recovered from RCSB-PDB^41^ and to fill in the missing loops and atoms, assign bond orders, the protein preparation module in MOE v2018.0101 was followed out and AMBER 10:EHT force field used for the handling of formal charges. The prepared structure was further used to establish the binding mode of the newly synthesized derivatives and by using the centroids of the residues Kcx-490,His-492, His-519,His-545, Asp-633, Ni-901 and Ni-902, the global search coordinates were generated. The Triangle Matcher was primary placement method, London dG was selected as placement scoring method. For the finesse of the initially brought forth 30 poses, Rigid Receptor protocol was employed.

#### In-silico ADME analysis

Physicochemical and pharmacokinetic properties (absorption, distribution, metabolism and excretion) of all the compounds **3(a–u)** were assessed by SwissADME web server. The SMILES format of each synthetic derivative was obtained from ChemDraw and analyze by the web server to predict the pharmacokinetic profile.

## Data Availability

All datasets on which the conclusions of the manuscript rely are presented in the paper.
